# Circulating exo-miR-154-5p regulates vascular dementia through endothelial progenitor cell-mediated angiogenesis

**DOI:** 10.3389/fncel.2022.881175

**Published:** 2022-07-29

**Authors:** Xue Han, Li Zhou, Yu Tu, Jiajia Wei, Jiajia Zhang, Guojun Jiang, Qiaojuan Shi, Huazhong Ying

**Affiliations:** ^1^Zhejiang Provincial Key Laboratory of Laboratory Animals and Safety Research, Hangzhou Medical College, Hangzhou, China; ^2^School of Pharmaceutical Sciences, Zhejiang Chinese Medical University, Hangzhou, China; ^3^Department of Pharmacy, Affiliated Xiaoshan Hospital, Hangzhou Normal University, Hangzhou, China

**Keywords:** vascular dementia, miR-154-5p, exosome, endothelial progenitor cells, angiogenesis

## Abstract

**Background:**

Vascular dementia (VaD) mainly results from cerebral vascular lesions and tissue changes, which contribute to neurodegenerative processes. Effective therapeutic approaches to targeting angiogenesis may reduce mortality of VaD. Endothelial progenitor cells (EPCs) play a key role in postnatal angiogenesis. Many exosomal microRNAs (exo-miRNAs) have been reported to involve in the development of dementia. The present study was designed to investigate whether the expression profile of the exo-miRNAs is significantly altered in patients with VaD and to reveal the function of differentially expressed miRNAs and the relevant mechanisms in EPC-mediated angiogenesis in VaD rat model.

**Results:**

Exosomes isolated from serum of patients with VaD (*n* = 7) and age-matched control subjects (*n* = 7), and miRNA sequencing and bioinformatics analysis found that circulating exosome miRNA-155-5p, miRNA-154-5p, miR-132-5p, and miR-1294 were upregulated in patients with VaD. The expression of miRNA-154-5p was further verified to be upregulated in clinical samples (*n* = 23) and 2-vessel occlusion-induced VaD rat model by reverse transcription quantitative PCR (RT-qPCR). Notably, miRNA-154-5p inhibition in bone marrow-EPCs (BM-EPCs) from VaD rats improved EPC functions, including tube formation, migration, and adhesion, and elevated concentrations of vascular endothelial growth factor (VEGF) and stromal cell-derived factor-1α (SDF-1α). The mRNA levels of ICAM-1, VCAM-1, and MCP-1 were reduced in miRNA-154-5p-inhibited EPCs. In addition, miRNA-154-5p inhibition increased the level of superoxide dismutase (SOD), and decreased reactive oxygen species (ROS) in EPCs. PRKAA2 was chosen as a promising target gene of miR-154-5p, and miRNA-154-5p inhibition upregulated the protein expression of AMPKα2. Furthermore, upregulation of miR-154-5p markedly diminished EPC functions and inhibited angiogenesis following EPC transplantation in VaD rats.

**Conclusion:**

Circulating exo-miR-154-5p was upregulated in patients with VaD, and miR-154-5p upregulation was associated with impaired EPC functions and angiogenesis in VaD rat model. Therefore, miR-154-5p is a promising biomarker and therapeutic strategy for VaD.

## Introduction

Vascular dementia (VaD) accounts for roughly 15–30% of dementia cases worldwide and increases linearly with age (Terracciano et al., 2021). VaD is characterized by progressive cognitive impairment, neurodegeneration, and memory difficulty, which is mainly attributed to cerebrovascular pathologies (Ghassab-Abdollahi et al., 2021). VaD is becoming the second most common cause of dementia next only to Alzheimer’s disease (AD). Although numerous studies have been made on the elucidation of mechanisms for VaD in recent years, the treatment approaches still focus on controlling the associated cardiovascular and cerebrovascular symptoms (Librizzi et al., 2021). There is still a lack of effective strategies against VaD. Therefore, investigation of the mechanism underlying cerebrovascular pathologies and further exploration of the novel therapeutic strategies have become a pivotal medical issue.

Emerging evidence suggests that exosomes play a vital role in the pathogenesis of neurodegeneration (Beatriz et al., 2021). Exosomes are generated and secreted through exocytosis of microvesicles (MVs) by mass of cell types, including stem cells (Fayazi et al., 2021). They are released into the extracellular space and exist in most body fluids, including saliva, plasma, urine, and even breast milk. These MVs can transfer nucleic acids and proteins to nearby cells, thereby affecting the activation of the recipient cells (Li et al., 2021). MicroRNAs (miRNAs) are conserved, endogenous, and small non-coding RNAs (22 nucleotides in length) that anneal to the 3′-UTR of their target mRNAs to negatively regulate the translation (Kong et al., 2021). Importantly, abnormal expression of miRNAs is associated with a wide range of disorders. It has been shown that miRNA expression pattern in exosome from peripheral blood implies a diagnostic biomarker for the stage and grade of liver diseases (Murakami et al., 2012). Moreover, exosomal miRNAs (exo-miRNAs) in cerebrospinal fluid has been reported to be specific and sensitive biomarkers for differentiating Parkinson’s disease (PD) from healthy and AD (Gui et al., 2015). However, alterations of miRNA content in serum exosome from patients with VaD have not yet been described.

It is well-established that VaD is featured in cerebrovascular dysfunction, which precedes neurodegeneration. Structural and functional lesion of cerebral blood vessels results in cerebral blood flow regulation disturbance, vascular reserve consumption, and a decrease in brain repair ability (Raz et al., 2016). Chronic cerebral hypoperfusion (CCH) begins with such cerebrovascular damage that will further disrupt the homeostasis, such as oxidative stress, inflammation, mitochondrial and neurotransmitter dysfunction, and apoptosis (Du et al., 2017). In recent years, studies suggest that endothelial progenitor cells (EPCs) have a potential therapeutic application for vascular diseases (Han et al., 2017a; Zhou et al., 2019). EPCs possess the properties of migrating and homing to the injury area to promote angiogenesis and vasculogenesis (Han et al., 2017b). In addition, EPCs can derive from bone marrow and release pro-angiogenic growth factors liking vascular endothelial growth factor (VEGF). Clinical research has reported that a reduction of circulating EPCs was positive correlated with the cognitive dysfunction in patients with AD (Kong et al., 2011). Transplantation of EPCs can prolong the lifespan in stroke-prone spontaneously hypertensive rats (SHR-SP) and promote local angiogenesis in ischemic brain (Peng et al., 2018). Notably, EPC-derived MVs were able to trigger neoangiogenesis *via* a horizontal transfer of mRNA to endothelial cells of micro-/macro-vasculature (Deregibus et al., 2007). In short, EPCs have promising therapeutic potential against vascular brain diseases. Nevertheless, the relation between EPC bioactivity and CCH-induced cognitive impairment remains unclear.

In the present study, we established a bilateral common carotid artery occlusion (BCCAO) rat model, which could imitate the pathogenesis of VaD (Jiwa et al., 2010). We aimed to explore the effect of differentially expressed exo-miRNAs on EPC-mediated angiogenesis in BCCAO rats.

## Materials and methods

### Clinical samples

This research was approved by the Ethics Committee of Zhejiang Xiaoshan Hospital (Hangzhou, China). A cohort of 30 patients with VaD at the Department of Neurology, Zhejiang Xiaoshan Hospital, and 12 healthy young and 22 healthy elder controls at the Department of Physical Examination Center, Zhejiang Xiaoshan Hospital were enrolled. All patients were Han Chinese and were recruited from November 2019 to January 2020. The patients with VaD were diagnosed by a neurologist based on the National Institute of Neurological Disorders and Stroke (NINDS)-Association Internationale pour la Recherche et I’Enseignement en Neurosciences (AIREN) clinical criteria (Román et al., 1993). Patients with VaD were scored with the clinical dementia rating (CDR), the Minimum Mental State Examination (MMSE) criteria and the Hachinski criteria (Table 1). The exclusion criteria were a history of a head injury with loss of consciousness, physical abnormalities, and meeting Diagnostic and Statistical Manual of Mental Disorders, the fourth edition (DSM-IV) criteria for substance dependence or current mood or anxiety disorders. In addition, patients administrated with anti-angiogenic medications for any indication were also excluded. All participants signed written informed consent.

**TABLE 1:** Primers used for RT-PCR analysis.

Gene	Species	Forward primer	Reverse primer
miR-155-5p	Human	5′-GTGTTAATGCTAA TCGTGATAGGGG-3′	5′-CAGTGCAGGG TCCGAGGTATT-3′
miR-154-5p	Human	5′-CCAGCGTGTAGG TTATCCGTG-3′	5′-CAGTGCAGGGTC CGAGGTATT-3′
miR-132-5p	Human	5′-CCGTGGCTTTC GATTGTTACT-3′	5′-CAGTGCAGGGTCC GAGGTATT-3′
miR-1294	Human	5′-AGCGTGTGTG AGGTTGGCAT-3′	5′-CAGTGCAGGG TCCGAGGTATT-3′
miR-154-5p	Rat	5′-AGGTTATCCGT GTTGCCT-3′	5′-GAACATGTCTG CGTATCTC-3′
ICAM-1	Rat	5′-AGATCATACGGGTT TGGGCTTC-3′	5′-TATGACTCGTGAAA GAAATCAGCTC-3′
VCAM-1	Rat	5′-TTTGCAAGAAA AGCCAACATGAAAG-3′	5′-TCTCCAACAGTT CAGACGTTAGC-3′
MCP-1	Rat	5′-GTCACCAAGCTC AAGAGAGAGA-3′	5′-AGTGGATGCAT TAGCTTCAGA-3′
β-actin	Rat	5′-AAGTCCCTCAC CCTCCCAAAAG-3′	5′-AAGCAATG CTGTCACCTTCCC-3′

### Blood sampling

Due to the time of blood drawing needed to accommodate the clinic and laboratory, all sample collection in this study were conducted between 9 a.m. and 11 a.m. Peripheral blood from all subjects was placed in a serum tube for 1 h, and the tubes were centrifuged at 3,000 × *g* for 10 min at 4°C. The supernatant was regarded as serum and stored at −80°C until detection.

### Exosome preparation

The procedure of exosome preparation as described before with minor modification (Li et al., 2017). In brief, serum was melted in 37°C water bath and supplemented with 10 times the volume of phosphate-buffered saline (PBS) buffer. After filtration with 0.22 μm filter membrane, the supernatant was centrifuged at 100,000 × *g* overnight at 4°C (Optima-L80XP, Beckman Coulter, CA, United States). The supernatant was discarded carefully, then appropriate amount of PBS was added and centrifuged at 100,000 × *g* for 2 h. The obtained precipitate was exosome. The exosome samples were suspended in 20–30 μl PBS, and the morphology was observed using the Tecnai transmission electron microscopy (FEI, OR, United States) under 80 kV. The particle size of exosomes was detected with a ZetaView Nanoparticle Tracking Analysis (Particle Metrix, Meerbusch, Germany).

### RNA sequencing

Differential gene expression was analyzed with RNA-sequencing assay. Exosome samples from healthy control and patients with VaD (*n* = 7 per condition) were used to isolate less than 200 nt RNA with miRNeasy Mini Kit (QIAGEN, Hilden, Germany). Sequencing libraries were built and transcriptome sequenced using a HiSeq 2000 (Illumina, San Diego, CA, United States). Agilent 2100 Bioanalyzer was chosen to analyze insert sizes in complementary DNA (cDNA) libraries. Gene expression data were evaluated on Dr. Tom system (BGI, Wuhan, China). Raw sequencing data are available in Supplementary material.

### Real-time quantitative PCR analysis

Total RNA from exosomes and tissues was extracted using TRIZOL reagent according to the manufacturer’s instruction (Takara) and was reverse-transcribed with a PrimeScript™ RT Reagent Kit (Takara). Real-time PCR was performed using SYBR Premix Ex Taq™ (Takara) with the LightCycler 480 System (Roche Diagnostics, Basel, Switzerland). Primer sequences were synthesized in Sangon Biotech (Shanghai, China) and listed in Table 1.

### Animals

Male Sprague-Dawley rats were 18-month-old supplied by the Laboratory Animal Center of Hangzhou Medical College (SPF grade; Hangzhou, China). All animals were housed at 22–25°C and 40–60% humidity under a 12-h light/dark cycle. Rats were with free access to food and water. Efforts were made to minimize their suffering. The study procedures were carried out according to the National Institutes of Health Guidelines for the Care and Use of Laboratory Animals. The study protocol was approved by the Ethics Committee of Laboratory Animal Care and Welfare, Hangzhou Medical College. After adaptive feeding for a week, rats were randomly divided into sham group and VaD group.

### Establishing of vascular dementia rat model

The bilateral common carotid artery occlusion (BCCAO) rat model has been widely accepted as an experimental model of VaD (Leardini-Tristão et al., 2017, 2020). The procedures were carried out as previously reported (Leardini-Tristão et al., 2017, 2020). In brief, after rats were anesthetized with phenobarbital sodium (40 mg/kg, *i.p.*), the bilateral common carotid arteries were carefully isolated from the peripheral vagus nerve through a midline incision and double-ligated with surgical silk. Sham group was subjected to the same procedure, except that the bilateral common carotid artery was not ligated. The rats were kept on a heating pad at 38°C until recovery from anesthesia.

### Cerebral blood flow monitoring

For cerebral blood flow monitoring, a laser speckle blood flow imaging system (SIM BFI-WF; SIM Opto-Technology Co., Ltd., Wuhan, China) was employed. The skin of the head was cut to expose the subdermal blood vessels. A thin circle of glass was inserted into the window frame, and cerebral blood flow velocity was detected before and after establishing of VaD rat model.

### Morris water maze

The Morris water maze (MWM) test was used to evaluate the spatial learning and memory of rats as previously reported with minor alteration (Xu et al., 2016). After 3 months of BCCAO operation, the rats began to place navigation test for 4 consecutive days. The pool was 1.2 m in diameter and height 0.6 m, and filled with water and opaque liquid maintained 24–26°C inside. The external visual reminder around the pool were kept constant, which helped for rats to spatial orientation. A platform (12 cm in diameter) was submerged 2.5 cm below the water surface in the northeaster quadrant. The animals received four trials per day. In each trial, rats were softly released into the water facing the tank wall at one of the four starting positions. The rats were permitted a maximum of 60 s to find the hidden platform. If the rats failed to find the hidden platform within the specified time, they were guided to the platform and allowed to stay for 15 s, and a score of 60 s was assigned. The escape latency was recorded to analyze the spatial learning. On the fifth day, a probe trial was carried out to assess spatial memory. Briefly, the rats were allowed to freely swim for 60 s in the pool from which the platform was removed, and the time spent in the target quadrant were recorded. The path taken by animals were monitored using a computer-based video camera, and the data was analyzed with the MT-200 image analyzing software (Taimeng Co., Chengdu, China).

### Nissl staining and immunofluorescence staining

Rats in both groups were sacrificed after the MWM test, and brain tissues were collected for pathological observation. Brain samples were incubated with 4% paraformaldehyde for 24 h at room temperature and were embedded in paraffin. After that, embedded brain paraffin sections were sliced into 5 μm section, then de-paraffinized with xylene and dehydrated with a graded concentration of ethanol. Nissl-stained sections were used to stain the section for 5 min. Representative pictures of Nissl-stained sections in the CA1, CA3, DG, and cortex regions were taken under a high-power optical microscope (Leica Microsystems, Wetzlar, Germany). The number of neurons was counted using ImageJ software (National Institutes of Health, Bethesda, MD, United States).

For immunofluorescence stain, angiogenesis in the brain tissues was detected using a monoclonal antibody CD31 (diluted 1:300; R&D System; Minneapolis, MN, United States), and Iba-1-positive microglial cells were evaluated using a rat antibody (diluted 1:200; Abcam, ab178846) followed by immunofluorescence staining using AlexaFluor 488 IgG antibody (Jackson ImmunoResearch; West Groove, PA, United States). Dapi (diluted 1:300; Beyotime, Shanghai, China) was used for nuclei staining. CD31-positive or Iba-1-positive stain were observed using a fluorescence microscope (Leica Microsystems, Wetzlar, Germany).

### TUNEL assay

Apoptotic neurons were measured using a TUNEL Assay Kit (Wuhan Boster Biological Technology, Ltd., Wuhan, China) according to the manufacturer’s instructions. Briefly, brain samples were incubated with anti-neuronal nuclei antibody overnight at 4°C. Sections were then washed three times with PBS and incubated with TUNEL reaction mixture for 1 h at room temperature. Images of TUNEL-stained sections in the cortex were captured using a fluorescence microscope. TUNEL-positive neurons were quantitated using ImageJ software.

### Activity of malondialdehyde, superoxide dismutase, and glutathione peroxidase

Serum from sham and VaD groups were collected for the measurement of oxidative stress-related biological parameters. The activities of malondialdehyde (MDA), superoxide dismutase (SOD), and glutathione peroxidase (GSH-PX) were determined with commercial kits according to the manufacturer’s instructions (Nanjing Jiancheng Bioengineering Institute, Nanjing, China). The samples were analyzed in duplicate.

### Measurement of inflammatory factors and myeloperoxidase

Brain tissue samples were analyzed for interleukin (IL)-1β, tumor-necrosis factor (TNF)-α, and myeloperoxidase (MPO) with commercial ELISA kits according to the manufacturer’s instructions (Anogen, Mississauga, ON, Canada). All readings were made from an ELISA plate reader. The samples were analyzed in duplicate.

### Estimation of bone marrow-endothelial progenitor cell functions

Isolation and culturing of BM-EPCs were performed as previously described (Han et al., 2017a). To evaluate the ability of angiogenesis, Matrigel tube formation assay was performed. After 7 days of culture, BM-EPCs were replated into a 96-well plate, which was precoated with Matrigel (BD Biosciences; Bedford, MA, United States) with a concentration of 30,000 per 100 μl. Plate was incubated for 4–6 h at 37°C, images of tube morphology were captured using a microscope. Tube formation was assessed by counting tube numbers.

The ability of migration was estimated using a modified Boyden chamber assay as previously reported (Yu et al., 2016). Briefly, BM-EPCs were plated with a concentration of 30,000 per 100 μl on the upper chamber (8 μm pores), and cell-free medium containing 50 ng/ml VEGF (Sigma-Aldrich, Darmstadt, Germany) was added to the lower chamber of a 24-well Transwell plate (Corning; Lowell, MA, United States). The plate was incubated for 24 h at 37°C, and cells were then fixed with 2% paraformaldehyde and stained with Hoechst 33258 (Beyotime). The images were taken with a microscope. The migrated cells were counted using ImageJ software.

The adhesion assay was applied to assess the EPC function. A total of 30,000 cells were plated into a 96-well plate per well, which was precoated with the mouse vitronectin (1 μg/ml; Sigma). Cells were incubated for 2 h at 37°C, and non-adherent cells were carefully removed with PBS. Adherent cells were fixed with 2% paraformaldehyde and stained with Hoechst 33258. The stained cells were then assessed using a fluorescence microscope and counted with ImageJ software.

### Measurement of vascular endothelial growth factor, stromal cell-derived factor-1α, and superoxide dismutase concentrations

The concentrations of VEGF and stromal cell-derived factor-1α (SDF-1α) in culture medium were detected using commercial ELSA Kits (R&D System). The level of SOD was measured using a SOD Quantification Kit (Beyotime). The procedures were performed according to the manufacturers’ protocols. All samples were measured in duplicate.

### Determination of reactive oxygen species generation

Intracellular reactive oxygen species (ROS) level was measured with the membrane-permeable dye dihydroethidium (DHE; Invitrogen; Carlsbad, CA, United States). After 7 days cultivation, BM-EPCs were plated into 96-well plate and stained with DHE (2 μM) for 1 h at room temperature. Cells were gently washed with PBS twice to remove excess probe, and images were taken with a fluorescence microscope.

### Treatment of bone marrow-endothelial progenitor cells *in vitro*

After 7 days of culture, BM-EPCs were transferred with adenovirus or Lipofectamine 3000 (Invitrogen) in M199 medium supplemented with 1% FBS for 36 h. The sequences of miR-154-5p inhibitor, negative control, and miR-154-5p mimics (GenePharma; Shanghai, China) were as follows (5′-3′): miR-154-5p inhibitor, CGAAGGCAACACGGAUAACCUA; negative control sense, UUCUCCGAA CGUGUCACGUTT; negative control antisense, ACGUGACACGUUCGG AGAATT; miR-154-5p mimics sense, UAGGUUAUCCGUGUUGCCUUCG; and miR-154-5p mimics antisense, AAGGCAACACGGAUAACCUAUU. After 36 h of quiescent culture, cells were used to functions assay and EPC transplantation assay.

### Endothelial progenitor cell transplantation assay

After 5 days of culturing, BM-EPCs were labeled with 5-bromo-2′-deoxyuridine (BrdU; Thermo Fisher Scientific; Waltham, MA, United States) as previously described (Peng et al., 2018). In brief, endothelial growth medium-2 (EGM-2; Cambrex Corp; East Rutherford, NJ, United States) was used to dilute BrdU-labeled reagent (1:100), and the mixture was filtered with a 0.2 μm filter and heated to 37°C. A total of 2 ml of EGM-2 containing BrdU was added to cells that were plated in a six-well plate. On day 7, cells were washed with PBS for three times and harvested with 0.125% trypsin. Sprague-Dawley rat EPCs (400,000 cells) were transplanted into VaD rat *via* the tail vein. After 7 days of a single injection of EPCs, rats were sacrificed, and brain tissues were collected for immunofluorescence staining.

### Statistical analysis

Data are expressed as mean ± SD. Statistical analysis was performed using one-way analysis of variance (ANOVA) with the Newman–Keuls multiple comparison test, and Student’s *t*-test for two group analysis. A value of *p* < 0.05 was considered to be statistically significant.

## Results

### Characterization of extracellular vesicles extracted from serum

**FIGURE 1:**
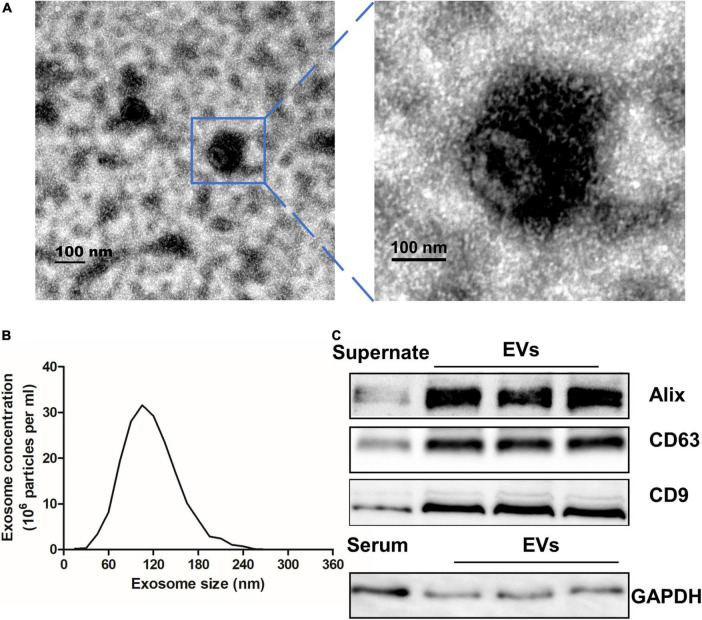
Characterization of EVs extracted from serum. **(A)** Transmission electron microscope was used to analyze extracted exosomes which were zoomed indicated by the blue frame. Scale bar = 100 nm. **(B)** The particle size of serum EVs was measured with a Nanoparticle Tracking Analysis. **(C)** EVs and Supernate or serum were detected by Western blot for the exosome-riched markers Alix, CD63, and CD9, and the negative marker GAPDH. EVs, extracellular vesicles; GAPDH, glyceraldehyde-3-phosphate dehydrogenase.

There were 52 participants included in this study, consisting of 30 (58%) patients with VaD and 22 (42%) healthy controls. The demographic and identity of these participators were summarized in Table 2. The extracellular vesicles derived from human serum were characterized using transmission electron microscopy, Nanoparticle Tracking Analysis, and Western blot assay. As shown in Figures 1A,B, the EVs were in the classical cup morphology of exosomes, and the particle size was in the range of 70–150 nm. Western blot analysis showed that the EVs expressed positive marker proteins of exosome, including Alix, CD63, and CD9, and did not express the glyceraldehyde-3-phosphate dehydrogenase (GAPDH), a negative marker of exosome (Figure 1C). The results indicate that the EVs purified from human serum are qualified exosomes.

**TABLE 2:** The demographic and identity of clinical samples.

Variables		Healthy controls	VaD	*P*
No of subjects		22	30	
Age		72.3 ± 11.7	75.3 ± 5.7	0.2241
Sex (M/F)		10/12	14/16	
**Clinical characteristics**				
CDR (%)	0.5–1	N/A	33.3%	
	2	N/A	40.0%	
	3	N/A	26.7%	
MMSE (%)	0–10	N/A	0.0%	
	10–24	N/A	100.0%	
Hachinski (%)	0–7	N/A	0.0%	
	≥7	N/A	100.0%	
Body Mass Index (kg/m^2^)		26.3 ± 2.5	26.6 ± 2.4	0.6956
Hypertension		8 (36.4%)	17 (56.7%)	0.1536
Diabetes		2 (9.1%)	7 (23.3%)	0.1868
Cardiovascular disease		3 (13.6%)	8 (26.7%)	0.2644

### miRNAs are differentially expressed in serum exosomes of patients with vascular dementia

**FIGURE 2:**
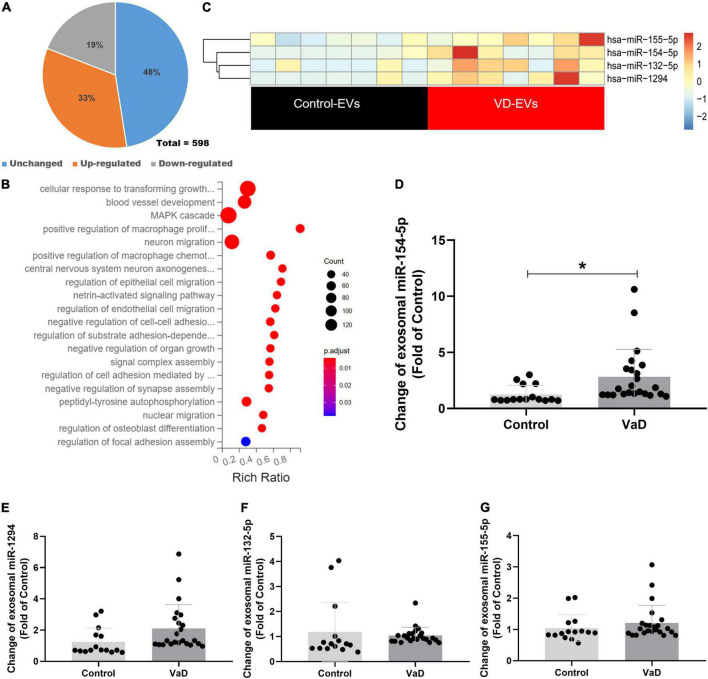
Bioinformatics analysis of miRNAs expression profiles in serum exosomes of patients with VaD. **(A)** Pie chart of differentially expressed miRNAs. The Jacinth part presented upregulated miRNAs; the gray part indicated downregulated miRNAs; the blue part presented unchanged miRNAs. **(B)** Heat map of differentially expressed miRNAs from patients with VaD and control group. **(C)** The KEGG analysis of the pathways enriched by the differentially expressed miRNAs from patients with VaD. **(D–G)** The upregulated miRNAs were further verified using RT-PCR in patients with VaD and control group (Control: *n* = 13, VaD: *n* = 23). *p < 0.05. Data are shown as mean ± SD. KEGG, Kyoto Encyclopedia of Genes and Genomes; RT-PCR, reverse transcription polymerase chain reaction; VaD, vascular dementia.

To explore whether the contents of exosomes were changed between patients with VaD and healthy controls, purify exosomes from seven normal subjects and seven patients with VaD who met the inclusion and exclusion criteria were selected to conduct miRNA library sequencing. Transcriptome analysis of miRNAs showed that a total of 598 mature miRNAs were detected, of which 33% were upregulated and 19% were downregulated both twice and more than twice high in VaD group compared with the control group (Figure 2A). Kyoto Encyclopedia of Genes and Genomes (KEGG) analysis showed that the genes targeted by these differentially expressed miRNAs were mainly enriched in blood vessel development, cellular migration, and cell adhesion signaling pathways (Figure 2B). This result indicates that the differentially expressed miRNAs may be involved in the regulation of cells, which possessed these biological functions. The miRNAs in VaD group that were upregulated by two times or more with statistical differences (*p* < 0.05) were chosen as the objects of study (Figure 2C), and specific change multiple is shown in Table 3. These 4 miRNAs were further verified in 15 normal subjects and 23 patients with VaD, and reverse transcription polymerase chain reaction (RT-PCR) results showed that miR-154-5p were significantly upregulated in the VaD group compared with the control group (*p* < 0.05, Figure 2D). Although there were no significant differences between control group and VaD group of miR-1294, miR-132-5p, and miR-155-5p (*p* > 0.05, Figures 2E–G). To further study the influence of aging on levels of miRNAs, 12 healthy young were recruited. The demographic and identity of healthy young and elder participators were summarized in Supplementary Table 1. There were no significant differences between young group and elder group for miR-154-5p, miR-1294, miR-132-5p, and miR-155-5p (*p* > 0.05, Supplementary Figure 1). These results suggest that miR-154-5p is confirmed to be significantly increased in serum exosomes of patients with VaD.

**TABLE 3:** The up-regulated miRNAs.

Name	Fold change (VaD/Ctrl)	*P*
Has-miR-154-5p	11.91	0.03
Has-miR-1294	8.76	0.02
Has-miR-132-5p	4.44	0.02
Has-miR-155-5p	2.81	0.02

### Establishing of vascular dementia rat model and upregulation of oxidative stress and inflammation in vascular dementia rats

**FIGURE 3:**
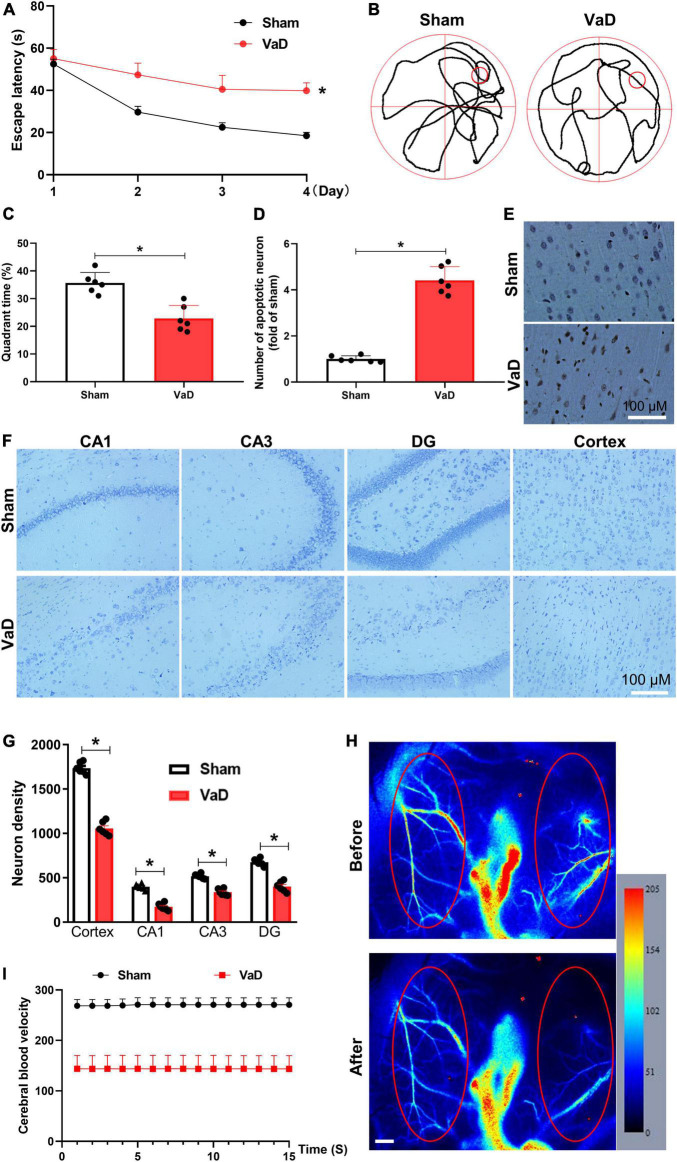
Establishing of VaD rat model. Sprague-Dawley rats were subjected to bilateral common carotid artery occlusion surgery to induce VaD rat model. Three months later, the Morris water maze (MWM) test was performed, then brains were collected for histopathological analysis. **(A)** The trajectory chart. **(B)** The latency time of rats to reach a hidden platform from day 1 to day 4 were recorded. **(C)** The percentage of time spent in the platform quadrant was calculated. **(D)** The statistical chart for TUNEL-positive cells. **(E)** Representative images of TUNEL staining in the cortex region. Representative images of Nissl staining in the CA1, CA3, DG, and cortex regions **(F)**, and neuron density **(G)**. **(H)** Cerebral blood velocity before and after surgery. Red ellipse represents region of interest. Scale bar, 0.5 mm. **(I)** The cerebral blood velocity was measured in regions of interest. [**(A–G)**: *n* = 6; **(H,I)**: *n* = 3]. *p < 0.05. Data are shown as mean ± SD.

To evaluate the influence of miR-154-5p on VaD in brain tissue, BCCAO surgery was performed to induce VaD model. After 3 months of surgery, spatial memory was assessed by the MWM test, and TUNEL stain and Nissl stain were used to evaluate pathologic changes in the cerebral tissues of VaD rats. In the acquisition trials, VaD rats exhibited an obvious impairment in spatial learning, which was evidenced by longer latency time to reach the platform compared with sham group (*p* < 0.05, Figure 3A). In the probe trial, the trajectory chart showed decreased times in platform site crossings in VaD rats (Figure 3B). The analysis showed that the time spent in the target quadrant was greatly reduced in VaD rats compared with sham group (*p* < 0.05, Figure 3C). Moreover, as showed in Figures 3D,E, the number of TUNEL-positive cells was significantly increased in the cortex region of the VaD rats compared with sham rats (*p* < 0.05). Nissl staining revealed a marked neuronal injure evidenced by the disappeared Nissl bodies, and shrunken and deepened staining of cell bodies in the CA1, CA3, DG, and cortex region of VaD rats when compared to sham rats (*p* < 0.05, Figures 3F,G). In addition, representative images of cerebral blood flow before and after establishment of VaD model are showed in Figure 3H. The redder the color, the faster the blood flow, and the blue color indicates slow blood flow. The cerebral blood velocity decreased by 46.8% following BCCAO surgery was compared with before surgery (Figure 3I). The above results indicating a VaD rat model successfully established.

Levels of oxidative stress and inflammation in VaD rats were evaluated. VaD rats showed significantly higher serum MDA level, and lower SOD and GSH-PX levels than sham group (*p* < 0.05, Supplementary Figures 2A–C). Furthermore, the VaD rats exhibited more remarkable elevation of IL-1β, TNF-α, and MPO in cerebral tissues than sham rats (*p* < 0.05, Supplementary Figures 2D–F). Meanwhile, increased Iba-1-positive cells were confirmed by immunofluorescence staining of cortex sections from VaD rats (*p* < 0.05, Supplementary Figures 2G,H). These results suggest that levels of oxidative stress and inflammation are significantly increased in VaD rats.

### miR-154-5p is upregulated in the hippocampus, cortex, and bone marrow-endothelial progenitor cell from vascular dementia rats

To examine the expression of miR-154-5p in brain tissues and cellular, RT-PCR was performed. The expression of miR-154-5p was significantly increased in the hippocampus, cortex, and BM-EPC from VaD rats (*p* < 0.05, Supplementary Figure 2I), whereas there were no significant differences in microglia and astrocyte (*p* > 0.05, Supplementary Figure 2I), suggesting that miR-154-5p is likely involved in the development of VaD *via* endothelium.

### Inhibition of miR-154-5p improves vascular dementia-induced endothelial progenitor cell dysfunction and excessive oxidative stress in bone marrow-endothelial progenitor cells

**FIGURE 4:**
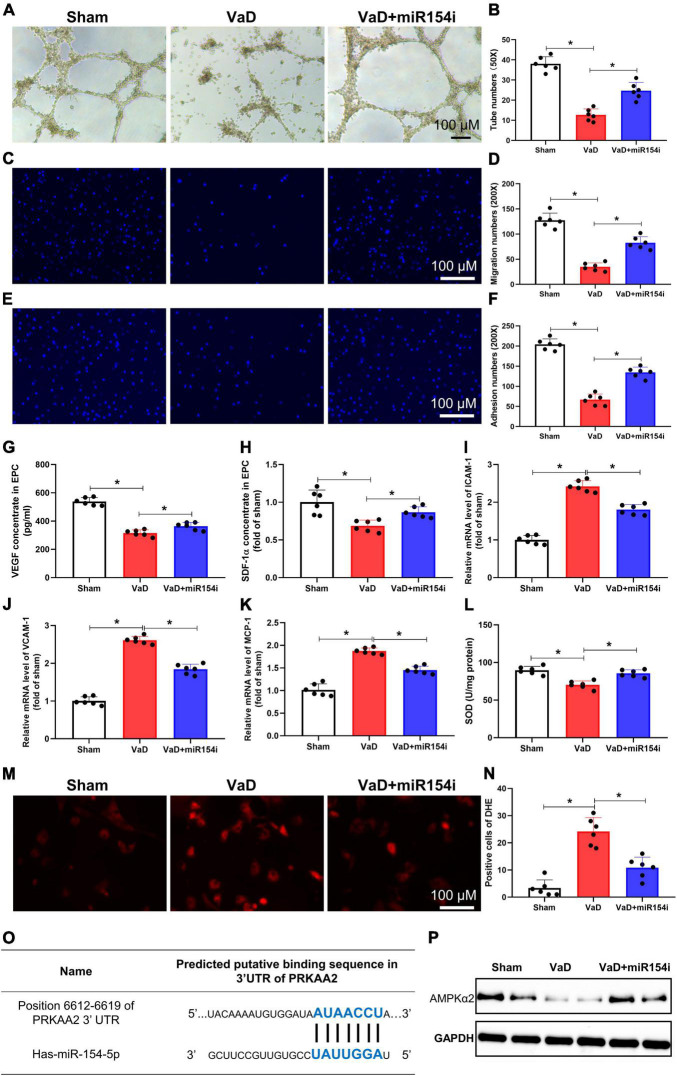
Inhibition of miR-154-5p improves VaD-induced EPC dysfunction and reduces oxidative stress in BM-EPCs. After 3 months of bilateral common carotid artery occlusion surgery, rats were euthanized, and BM-EPCs were harvested for functional tests. BM-EPC function was estimated by tube formation assay **(A,B)**, migration ability **(C,D)**, and adhesion capacity **(E,F)**. MiR-154-5p inhibitor significantly improved VaD-induced EPC dysfunction. The levels of VEGF **(G)** and SDF-1α **(H)** in culture medium were detected by ELISA kits. Results from the real-time PCR experiments for analysis the expression of ICAM-1 **(I)**, VCAM-1 **(J)**, and MCP-1 **(K)** in BM-EPCs. **(L)** SOD level in BM-EPCs was measured with a kit. Images of DHE staining **(M)** and quantitative analysis of DHE-positive cells **(N)**. **(O)** The predicted miR-154-5p-binding sites in PRKAA2 mRNA 3′-UTR were confirmed. **(P)** The downregulated level of AMPKα2 was increased by miR-154-5p inhibitor in BM-EPCs. 50×: scale bar, 100 μm; 200×: scale bar, 100 μm [**(A–N)**: *n* = 6; **(P)**: *n* = 3]. *p < 0.05. Data are shown as mean ± SD. BM-EPC, bone marrow-endothelial progenitor cell; SDF, stromal cell-derived factor; SOD, superoxide dismutase; VaD, vascular dementia.

Strong evidence demonstrates that cerebrovascular dysfunction links with VaD (Kalaria, 2018). Prior studies have reported that EPCs contribute to cerebral vascular repair disorders, such as AD and stroke (Bai et al., 2015; Zhang et al., 2018). To define the effect of miR-154-5p on EPC function in VaD rats, miR-154-5p inhibitor was used to knock down its expression. BM-EPCs were isolated from the VaD rats, and tube formation, migration, and adhesion ability were evaluated *in vitro*. BM-EPCs from VaD rats resulted in a significant decrease in the number of tubes (*p* < 0.05, Figures 4A,B), migration cells (*p* < 0.05, Figures 4C,D), and adhesion ability (*p* < 0.05, Figures 4E,F) compared with EPCs from sham group. miR-154-5p inhibitor significantly elevated tube formation, migration, and adhesion (*p* < 0.05, Figures 4A–F). In notice, miR-154-5p inhibitor markedly attenuated VaD-induced reduction of VEGF and SDF-1α concentrations in EPC from VaD rats (*p* < 0.05, Figures 4G,H).

Interestingly, miR-154-5p also potentially regulated adhesion molecules and oxidative stress. RT-PCR analysis revealed upregulated gene level expression of adhesion molecules, including ICAM-1, VCAM-1, and MCP-1, in BM-EPCs from VaD rats, whereas the miR-154-5p inhibitor significantly blunted the levels of these genes in BM-EPCs (*p* < 0.05, Figures 4I–K). Moreover, EPC from VaD rats showed a significant decrease of SOD, and miR-154-5p inhibitor dramatically increased the concentration of SOD in BM-EPCs (*p* < 0.05, Figure 4L). In agreement with this result, the miR-154-5p inhibitor effectively reduced production of ROS, as shown by DHE staining in EPC from VaD rats (*p* < 0.05, Figures 4M,N). In addition, we speculated the promising targets of miR-154-5p using miRDB, TargetScan 8.0, and miRNA.org PRKAA2, which could encode AMPKα2 was chosen as a potential target gene of miR-154-5p (Figure 4O). To prove that miR-154-5p could target AMPKα2, the protein expression of AMPKα2 was measured with Western blot. As shown in Figure 4P, the protein expression of AMPKα2 was remarkedly decreased in BM-EPCs from VaD rats compared with EPCs from sham group. The miR-154-5p inhibitor increased expression of AMPKα2 in BM-EPCs (Figure 4P). Taken together, these data indicate that inhibition of miR-154-5p was able to ameliorate EPC dysfunction in VaD rats, which may be associated with downregulated levels of adhesion molecules, oxidative stress, and AMPKα2 expression.

### Overexpression of miR-154-5p impairs endothelial progenitor cell functions and inhibits angiogenesis following endothelial progenitor cell transplantation in vascular dementia rats

**FIGURE 5:**
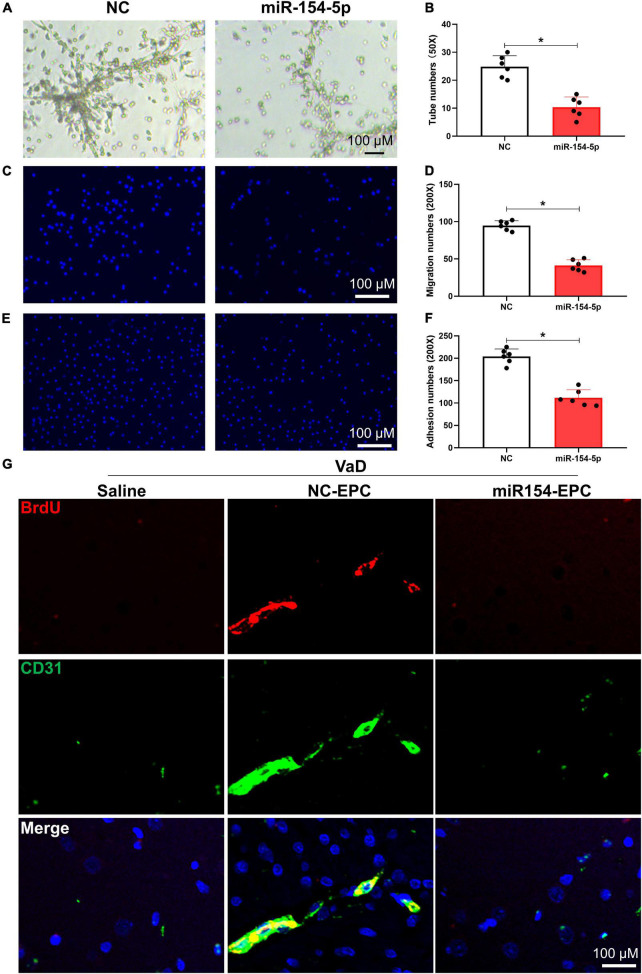
Overexpression of miR-154-5p impairs EPC functions and inhibits angiogenesis following EPC transplantation in VaD rats. After 7 days culture, BM-EPCs were transfected with normal control (NC) scrambled RNA or miR-154-5p mimics, and EPC functions were then evaluated using tube formation assay **(A,B)**, migration ability **(C,D)**, and adhesion capacity **(E,F)**. **(A)**: 50×: scale bar, 100 μm; **(C,E)**: 200×: scale bar, 100 μm. VaD rats were subjected to a single injection of saline or BrdU-labeled EPCs, which were transfected with NC RNA and miR-154-5p mimics. Seven days later, brain samples were harvested and stained with CD31 (green) and BrdU (red). **(G)** Representative images in the cortex region of VaD rats. 200×: scale bar, 100 μm [**(A–F)**: *n* = 6; **(G)**: *n* = 3]. *p < 0.05. Data are shown as mean ± SD. BrdU, 5-bromo-2′-deoxyuridine; EPC, endothelial progenitor cell; VaD, vascular dementia.

To further clarify the function of miR-154-5p on angiogenesis in VaD rats, we overexpressed miR-154-5p with RNA mimics in BM-EPCs. The overexpression of miR-154-5p alone markedly worsened EPC functions, reflected by reduced number of tubes, migration cells, and adhesion cells in rats compared with normal control group (*p* < 0.05, Figures 5A–F). Later, VaD rats were transplanted with BrdU-labeled EPCs, which were pretreated with miR-154-5p mimics or negative control, and immunofluorescent staining for endothelial cell-specific marker CD31 (green), followed by BrdU (red) were performed in the cortex of brain tissues. As shown in Figure 5G, some BrdU-positive cells were integrated into CD31-positive vessels in the cortex of VaD rats that received NC-EPC transplantation. However, the miR-154-5p-EPC transplantation showed little incorporation into cerebral angiogenesis of VaD rats (Figure 5G). Based on these results, miR-154-5p was sufficient to trigger poor angiogenesis in the brain of VaD rats.

## Discussion

The primary findings of the current study were as follows. First, miR-154-5p was significantly increased in serum exosomes from patients with VaD. Second, the expression of miR-154-5p was also upregulated both in brain tissues and in BM-EPCs, and inhibition of miR-154-5p markedly ameliorated EPC dysfunction, evidenced by reduced expression of adhesion molecule and diminished oxidative stress in BCCAO-induced VaD rats. Finally, we showed that upregulation of miR-154-5p obviously worsened EPC function and inhibited angiogenesis following EPC transplantation in VaD rats. Our results confirmed that miR-154-5p may be one of the important regulators of EPC-mediated angiogenesis participating in the development of VaD.

Vascular dementia is proposed to be a chronic cerebrovascular syndrome resulted from diverse etiologies, with cerebral vascular damage being the key pathological manifestation. The pathogenesis of VaD has not yet been elucidated and mainly includes injury of the cholinergic system, neuroinflammation, synaptic plasticity, and the toxicity of excitatory amino acids and free radicals (Kalaria et al., 2016). Majority of patients with VaD do not have any symptoms in the stage of onset, thus effectively diagnostic biomarker and therapeutic prevention seem to be quite important. Recently, many exciting evidences indicate that exosomal contents, which cause dramatically changes under neurological damage condition, represent a possibility for the diagnosis and treatment of neurodegenerative disorders (Yuyama and Igarashi, 2017; Hamlett Ledreux et al., 2018). In general, exosomes are a kind of secreted bilayer-enclosed MVs with a size range of 50–150 nm diameter (Hamlett Ledreux et al., 2018). In this study, our results showed that isolated MVs from human serum corresponded to properties of exosomes in morphology and size.

Exosomes can deliver important proteins, miRNAs, and short-interfering RNA during intercellular communication. They are considered as carriers for functional miRNAs binding to the enzyme Argonaute 2 and transferring to the surrounding cells, including neurons and astrocytes (Hamlett Goetzl et al., 2017). Notably, altered miRNAs profiles in CSF or blood exosomes were closely associated with neurodegenerative disorders. It has reported that decreased miR-193b in CSF exosomes could function in the progression of AD (Liu et al., 2014). Besides, Application of low-level IFNγ released exosomes containing miRNAs species is able to increase myelination, reduce oxidative stress, and improve remyelination in acute lysolecithin-induced demyelination (Pusic et al., 2014). However, the alteration in exosome-mediated miRNA signature in VaD remains further research to clarify, and it may be a possible disease pathology. Our results showed that miR-154-5p was significantly elevated in serum exosomes of patients with VaD compared with healthy control *via* bioinformatics analysis. Meanwhile, this increment was further conformed in the brain tissues and BM-EPC of VaD rats, implying miR-154-5p involved in the development of VaD.

For the Kyoto Encyclopedia of Genes and Genomes (KEGG) analysis, the different genes were mainly enriched in blood vessel development, cellular migration, and cell adhesion signaling pathways. Numerous studies have revealed that recruitment EPCs into damaged tissues is one of the key events in neovascularization (Bai et al., 2015; Pías-Peleteiro et al., 2017). Due to their distinct capabilities, EPCs possess a promising potential in treatment of vascular diseases. In a diabetic ischemic stroke model, transplanted EPCs contribute to improve stroke outcome, promote angiogenesis, and elevate VEGF expression (Bai et al., 2015). Unfortunately, EPC function was significantly undermined in patients with AD and VaD (Kong et al., 2011). In line with these results, we found that EPC functions, including tube formation, migration ability, and adhesion capacity, were impaired, and pro-angiogenic growth factors VEGF and SDF-1α were reduced in BCCAO induced for 3 months model. Moreover, EPCs have been shown to provide an abundant source of exosomes suggesting one of the pivotal components modifying the bioactivity. The exosomes-mediated transfer of miR-126 has been shown to prevent microvascular dysfunction and effectively ameliorate sepsis outcomes in mice (Zhou et al., 2018). miR-154-5p, as a conserved miRNA in mammal, is associated with cell proliferation, cardiac fibroblasts, cardiomyocyte hypertrophy, and oxidant stress (Sun et al., 2016; Wang et al., 2019). However, the role of miR-154-5p in cerebrovascular remains unknown. Here, we found that inhibition of miR-154-5p improved the BCCAO-induced EPC dysfunction, upregulation of adhesion molecule, and excessive oxidative stress level. As expected, overexpression of miR-154-5p could worsen the EPC functions *in vitro*.

The EPC transplantation has a powerful potential in vascular repair and revascularization in neurological disorders besides AD (Peng et al., 2018; Zhang et al., 2018). It is also a broad-spectrum method, and patients with different risk factors of neurodegeneration, including aging, diabetes, hypertension, etc., may be applicable. In APP/PS1 transgenic mice, the EPC transplantation markedly improved spatial learning and memory functions, repaired blood–brain barrier tight junction function, and stimulated angiogenesis (Zhang et al., 2018). The EPC delivery might also represent an advanced strategy for interventional treatment of VaD. In this study, the EPC transplantation in CCH-induced rats was helpful for angiogenesis in the cortex tissues. While transplanted the EPCs which were overexpressed miR-154-5p reversed the angiogenesis effect.

There is a limitation in this study. The mechanism that related to miR-154-5p regulation of EPC-mediated angiogenesis under pathological condition of VaD mainly confirmed in VaD rats, we did not verify in patients with VaD. This limitation could cause bias in our study. Nonetheless, these data on the upregulation of circulating exo-miR-154-5p in patients with VaD are intriguing. Obviously, the results need to be further evaluated in patients with VaD in the future.

## Conclusion

In conclusion, in the current study, we show that exosomal miR-154-5p expression is significantly increased in VaD individuals. The upregulation of miR-154-5p is sufficient to trigger the EPC dysfunction and impairment of angiogenesis. Inhibition of miR-154-5p has beneficial effects on ameliorating EPC functions implying the clinical potential of miR-154-5p inhibition during VaD therapy.

## Data availability statement

The datasets presented in this study can be found in online repositories. The name of the repository and accession number can be found below: National Center for Biotechnology Information (NCBI) BioProject, https://www.ncbi.nlm.nih.gov/bioproject/, PRJNA833586.

## Ethics statement

The studies involving human participants were reviewed and approved by the Ethics Committee of Zhejiang Xiaoshan Hospital. The patients/participants provided their written informed consent to participate in this study. The animal study was reviewed and approved by the Ethics Committee of Laboratory Animal Care and Welfare, Hangzhou Medical College.

## Author contributions

HY, XH, and QJS contributed to the literature search and study design. XH participated in the drafting of the manuscript. XH, LZ, YT, JW, and JZ carried out the experiments. XH and LZ contributed to data collection and analysis. All authors contributed to the article and approved the submitted version.
