# Supplementary material for: Circulating exo-miR-154-5p regulates vascular dementia through endothelial progenitor cell-mediated angiogenesis
Source: Front Cell Neurosci. 2022 Jul 29;16:881175. doi: 10.3389/fncel.2022.881175 (PMC9372489; doi:10.3389/fncel.2022.881175)
Supplement: Supplementary file 1 [file Data_Sheet_3.DOCX]

**Supplementary material**

**Circulating Exo-miR-154-5p Regulates** **Vascular Dementia through EPC-Mediated Angiogenesis**

*Xue Han^1,*^, Li Zhou^2,*^, Yu Tu^1^, Jiajia Wei^1^, Jiajia Zhang^1^, Guojun Jiang^3^, Qiaojuan Shi^1,#^, Huazhong Ying^1,2,#^*

*^1^ Zhejiang Provincial Key Laboratory of Laboratory Animals and Safety Research, Hangzhou Medical College, Hangzhou, China;*

*^2^ College of Pharmaceutical Science, Zhejiang Chinese Medical University, Hangzhou, China;*

*^3^ Department of Pharmacy, Affiliated Xiaoshan Hospital, Hangzhou Normal University, Hangzhou, China*


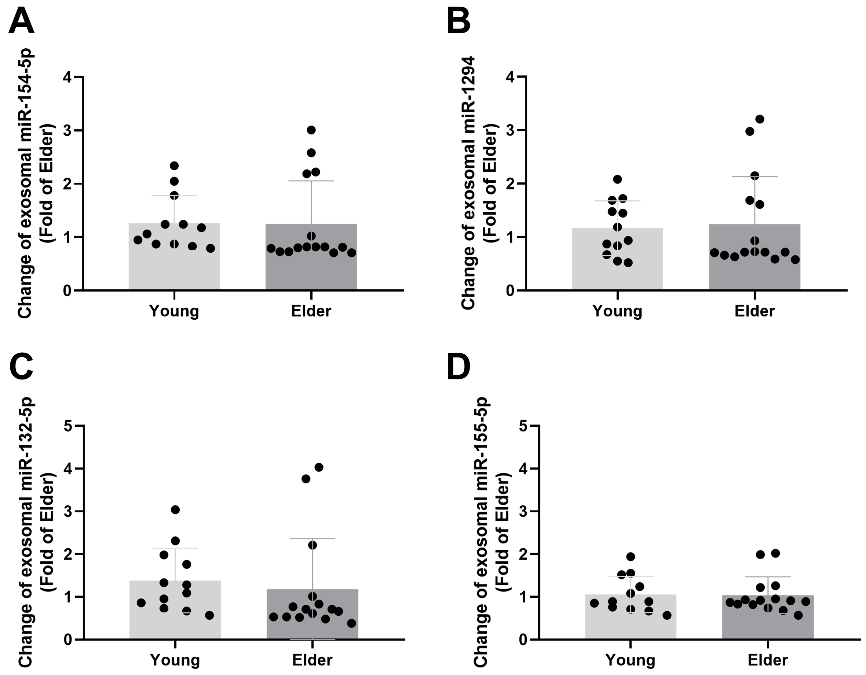


**Supplementary Figure S1** The expression of miRNAs in healthy young and elder group. (A-D) The levels of miRNAs were verified using RT-PCR in healthy young and elder group. (Young: n = 12; Elder: n = 15). Data are shown as mean ± SD.


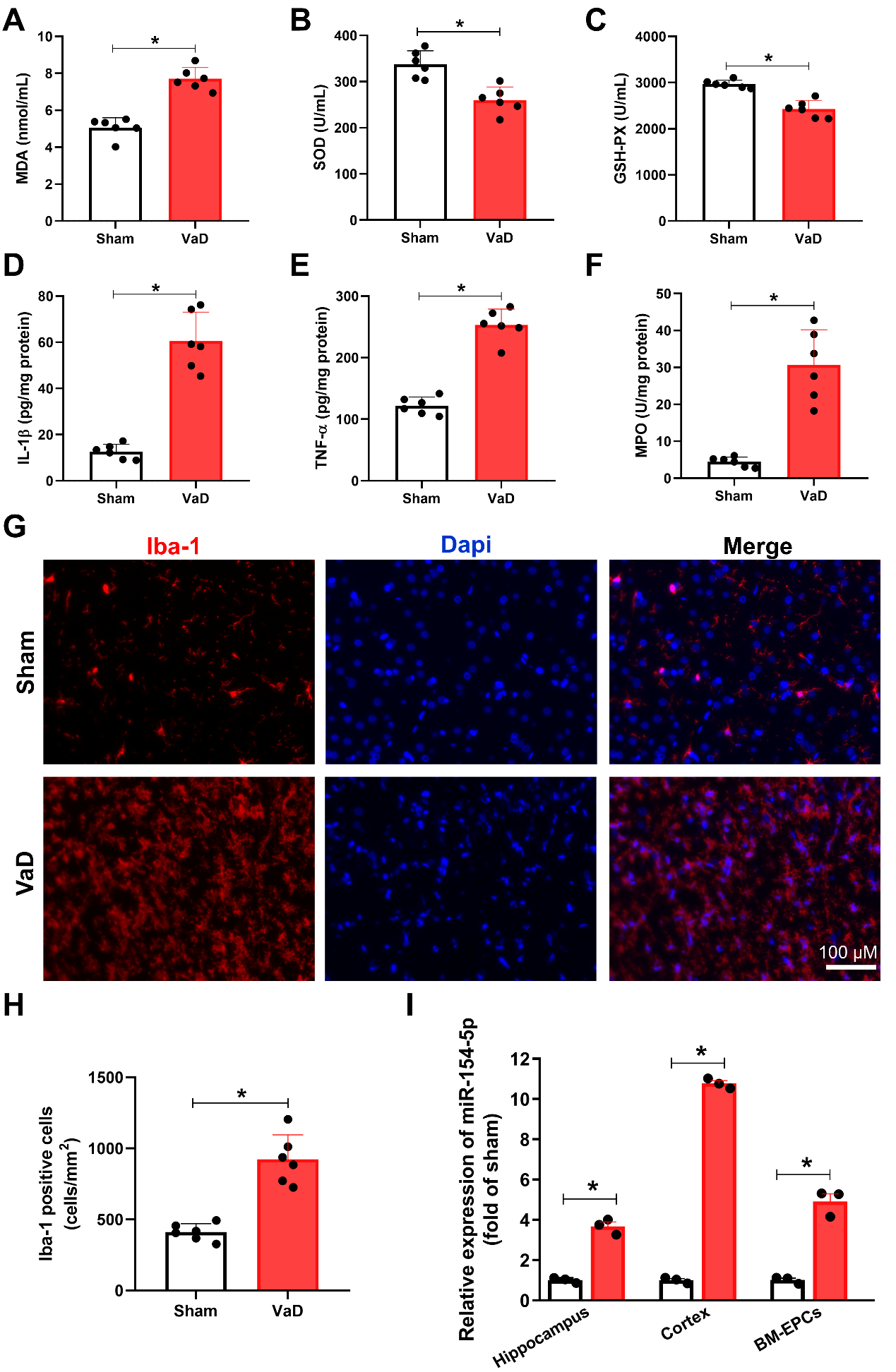


**Supplementary Figure S2** The levels of oxidative stress and inflammation in VaD rats. Serum levels of MDA (A), SOD (B), and GSH-PX (C) were measured in experimental animals. The inflammatory factors of IL-1β (D), TNF-α (E), and MPO (F) were detected by ELISA kits. (G) Representative immunofluorescent staining in the cortex of VaD rats. Sections were stained with Iba-1 (red) and Dapi (blue). (H) Iba-1 positive cells in the cortex were quantified. (I) The mRNA expression of miR-154-5p in cerebral hippocampus, cortex, and BM-EPCs in VaD rats. (A-H: n = 6; I: n = 3). **P* < 0.05. Data are shown as mean ± SD.

**Supplementary TABLE S1** The demographic and identity of clinical samples

| **Variables** | **Young** | **Elder** | ***P*** |
| --- | --- | --- | --- |
| No of subjects | 12 | 22 |  |
| Age | 27.3 ± 3.8 | 72.3 ± 11.7 | 0.0005 |
| Sex (M/F) | 7/5 | 10/12 |  |
| Body Mass Index (kg/m^2^) | 23.1 ± 2.6 | 26.3 ± 2.5 | 0.8964 |
| Hypertension | 1 (8.3%) | 8 (36.4%) | 0.0713 |
| Diabetes | 1 (8.3%) | 2 (9.1%) | 0.9873 |
| Cardiovascular disease | 1 (8.3%) | 3 (13.6%) | 0.5082 |
