# Supplementary figures and images for: Circulating exo-miR-154-5p regulates vascular dementia through endothelial progenitor cell-mediated angiogenesis
Source: Front Cell Neurosci. 2022 Jul 29;16:881175. doi: 10.3389/fncel.2022.881175 (PMC9372489; doi:10.3389/fncel.2022.881175)

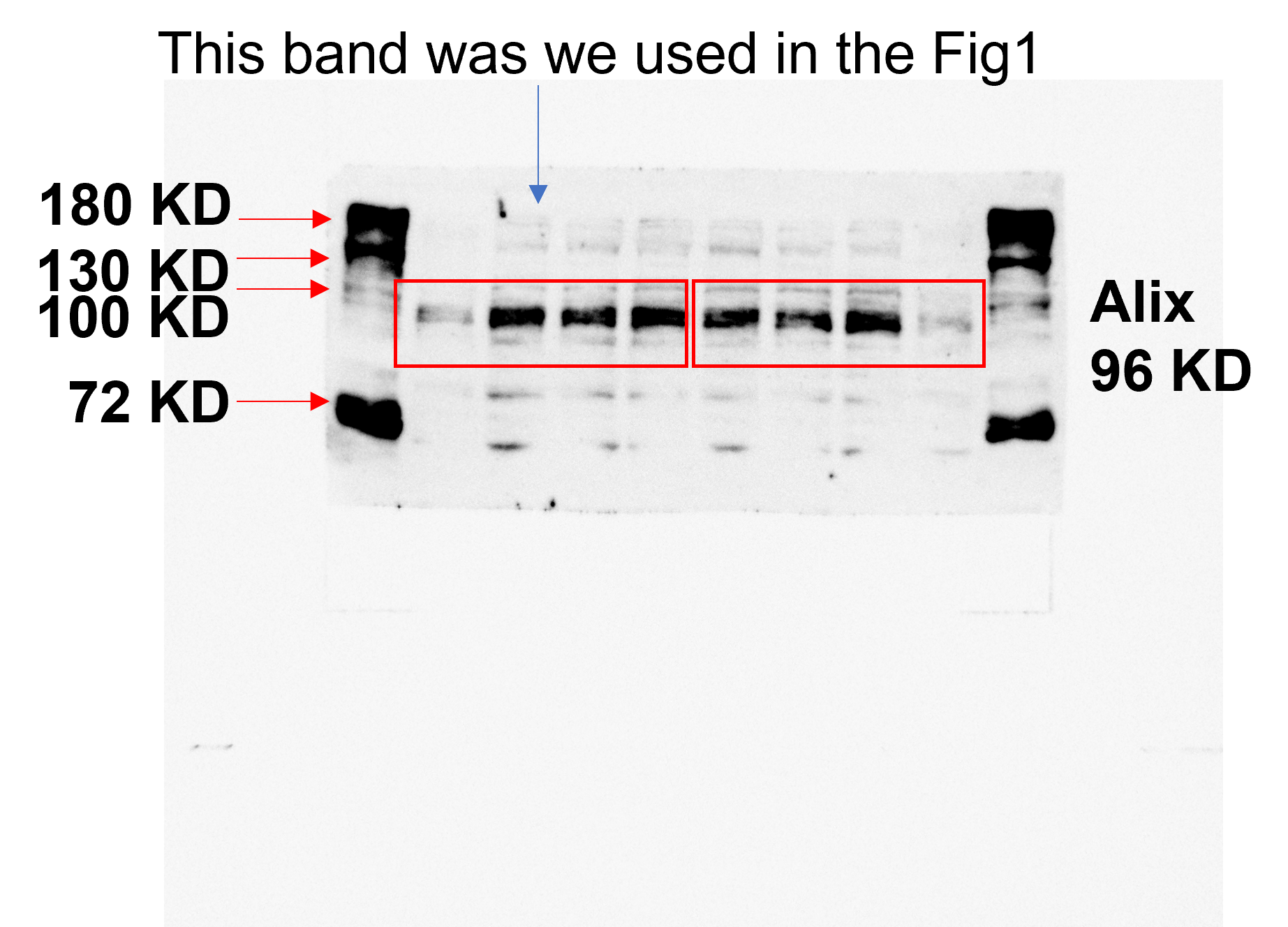

Supplement: Supplementary file 3 [file Data_Sheet_5.ZIP › Alix-band.tif]

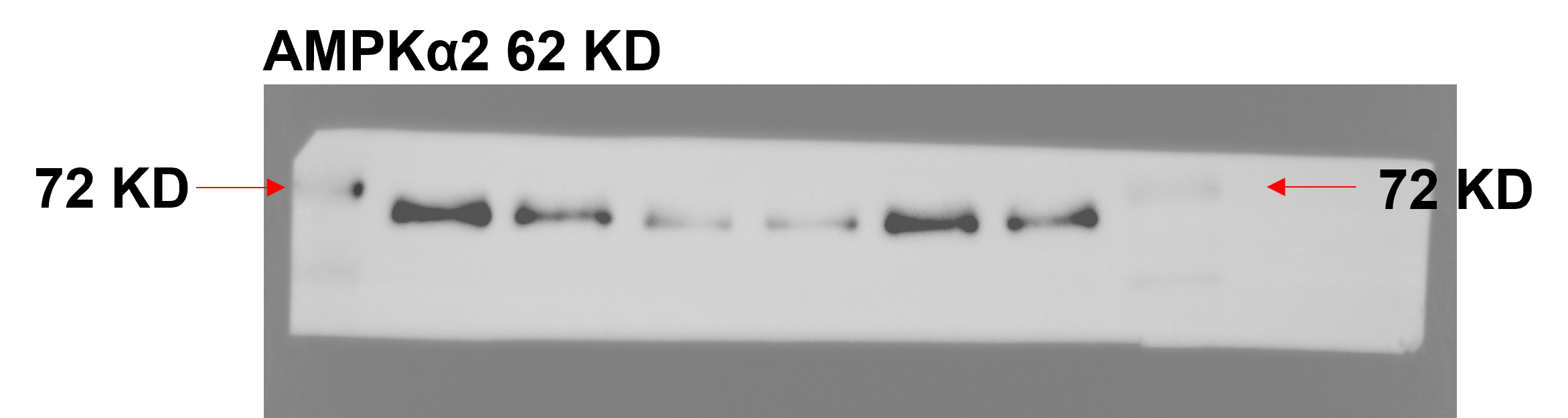

Supplement: Supplementary file 3 [file Data_Sheet_5.ZIP › AMPKa2-band.tif]

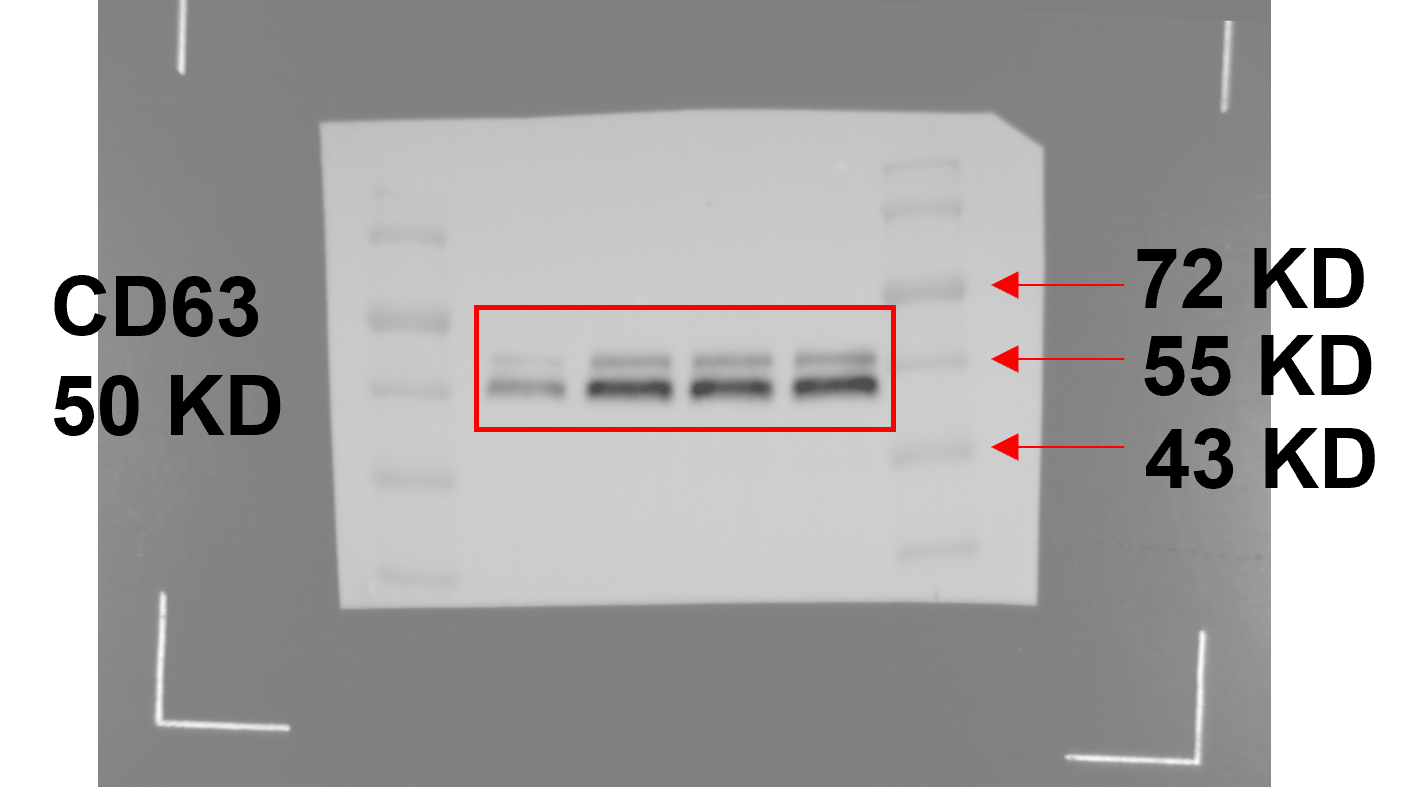

Supplement: Supplementary file 3 [file Data_Sheet_5.ZIP › CD63-band.tif]

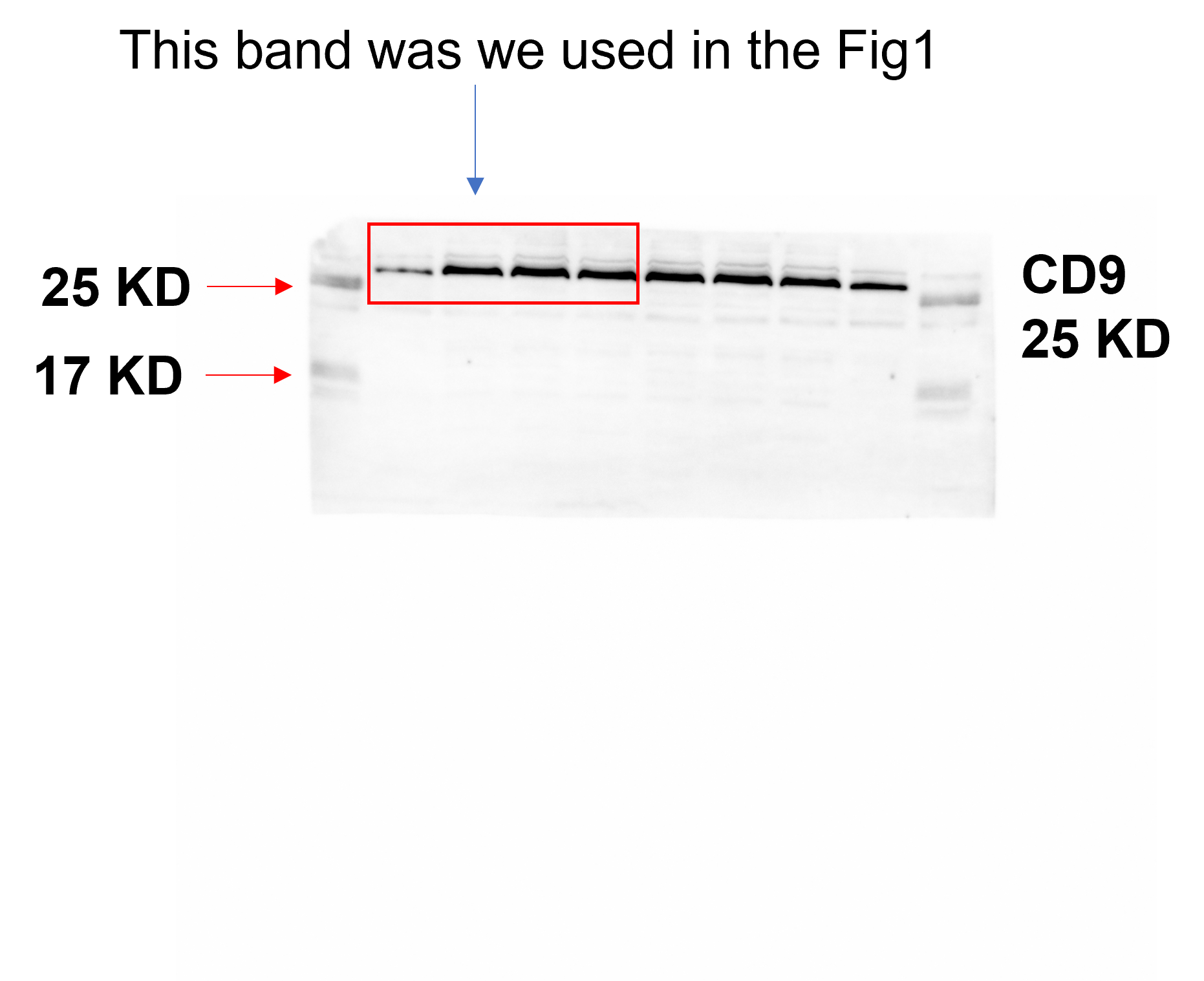

Supplement: Supplementary file 3 [file Data_Sheet_5.ZIP › CD9-band.tif]

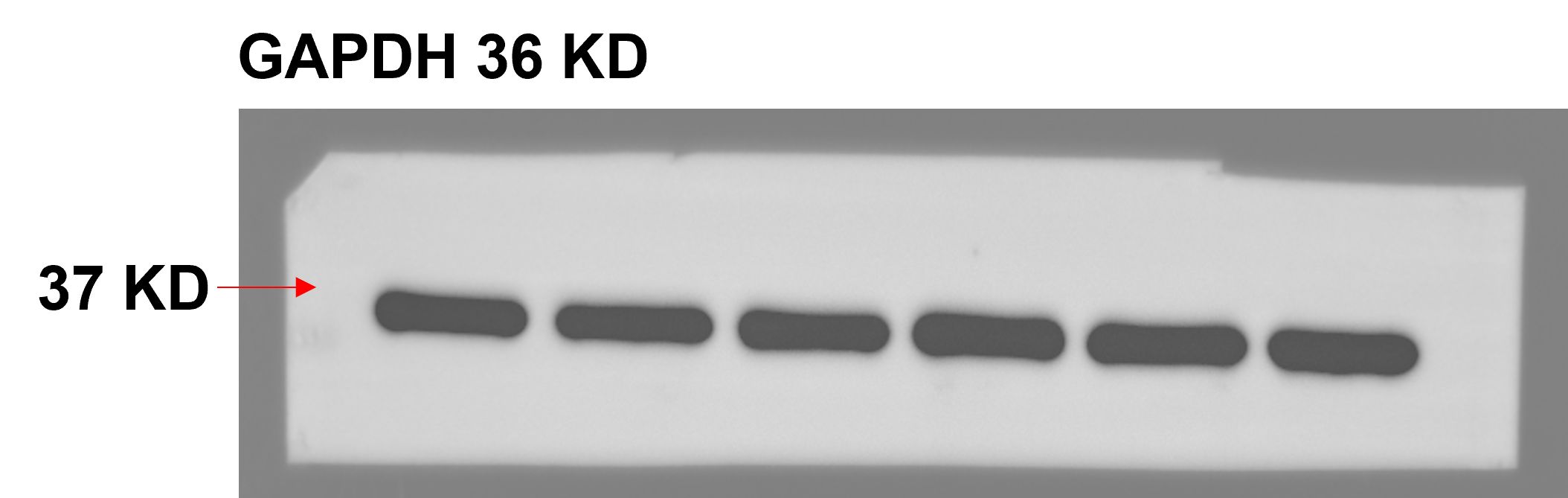

Supplement: Supplementary file 3 [file Data_Sheet_5.ZIP › GAPDH for AMPKa2-band.tif]

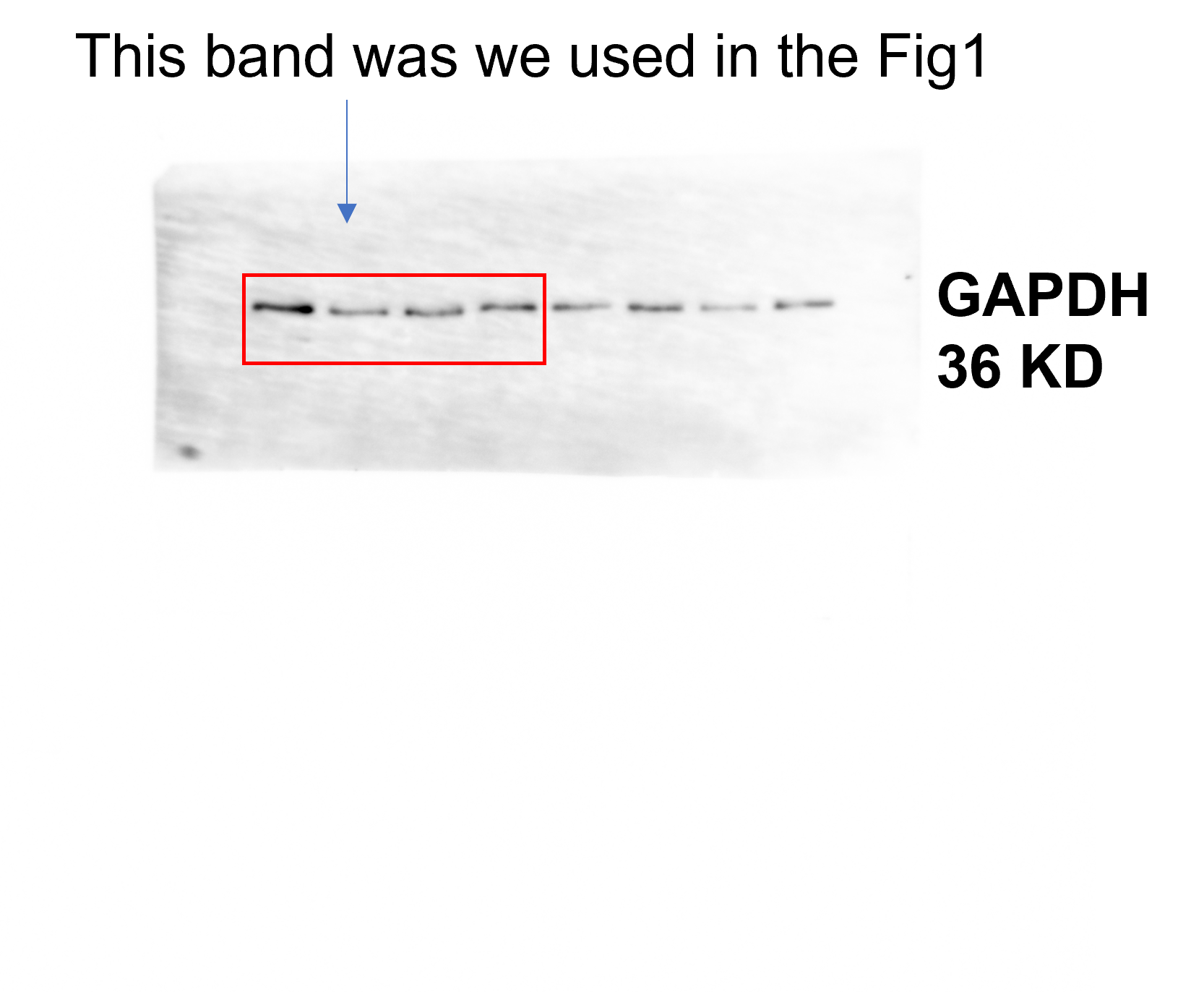

Supplement: Supplementary file 3 [file Data_Sheet_5.ZIP › GAPDH-band.tif]
